# Accurate and efficient protein embedding using multi-teacher distillation learning

**DOI:** 10.1093/bioinformatics/btae567

**Published:** 2024-09-24

**Authors:** Jiayu Shang, Cheng Peng, Yongxin Ji, Jiaojiao Guan, Dehan Cai, Xubo Tang, Yanni Sun

**Affiliations:** Department of Information Engineering, The Chinese University of Hong Kong, Hong Kong (SAR), HKG, China; Department of Electrical Engineering, City University of Hong Kong, Hong Kong (SAR), HKG, China; Department of Electrical Engineering, City University of Hong Kong, Hong Kong (SAR), HKG, China; Department of Electrical Engineering, City University of Hong Kong, Hong Kong (SAR), HKG, China; Department of Electrical Engineering, City University of Hong Kong, Hong Kong (SAR), HKG, China; Department of Electrical Engineering, City University of Hong Kong, Hong Kong (SAR), HKG, China; Department of Electrical Engineering, City University of Hong Kong, Hong Kong (SAR), HKG, China

## Abstract

**Motivation:**

Protein embedding, which represents proteins as numerical vectors, is a crucial step in various learning-based protein annotation/classification problems, including gene ontology prediction, protein–protein interaction prediction, and protein structure prediction. However, existing protein embedding methods are often computationally expensive due to their large number of parameters, which can reach millions or even billions. The growing availability of large-scale protein datasets and the need for efficient analysis tools have created a pressing demand for efficient protein embedding methods.

**Results:**

We propose a novel protein embedding approach based on multi-teacher distillation learning, which leverages the knowledge of multiple pre-trained protein embedding models to learn a compact and informative representation of proteins. Our method achieves comparable performance to state-of-the-art methods while significantly reducing computational costs and resource requirements. Specifically, our approach reduces computational time by ∼70% and maintains ±1.5% accuracy as the original large models. This makes our method well-suited for large-scale protein analysis and enables the bioinformatics community to perform protein embedding tasks more efficiently.

**Availability and implementation:**

The source code of MTDP is available via https://github.com/KennthShang/MTDP

## 1 Introduction

Protein characterization and annotation provide the foundational knowledge necessary to unravel the complex mechanisms underlying many biological processes. However, the complexity and variability of protein sequences pose significant challenges to traditional analysis methods, which struggle to capture their intricate patterns and relationships. Recently, deep learning-based algorithms have demonstrated remarkable success in protein data analysis, leveraging their ability to learn complex patterns and relationships from large datasets to predict gene ontology, 2D-/3D structure, and protein stability (Thumuluri *et al.* 2022, Fang *et al.* 2023, Hwang *et al.* 2024). An essential requirement for these successful applications is the representation of protein sequences as fixed-length numerical vectors, which encapsulate sequence composition information, structural signatures, and evolutionary relationships. Protein embedding models, which aim to extract informative and effective representations of proteins, play a crucial role in this process.

Recent advances in protein embedding models, which leverage unsupervised learning strategies such as masked language modeling, have yielded promising results in various bioinformatics applications (Elnaggar *et al.* 2021, Rives *et al.* 2021). However, as researchers strive to improve performance, these models have become increasingly large and complex, with millions or even billions of parameters, leading to a significant increase in computational resources required, particularly high-performance graphics processing units (GPUs), and often necessitating lengthy running times. For example, processing a ∼1 MB FASTA amino acids (FAA) file using a large model on a 24 GB memory GPU card can take approximately 7 h, thereby hindering research progress. In addition, the rapid growth of bioinformatics data containing sheer amount of protein sequences further exacerbates these issues. Thus, there is a growing demand for efficient protein embedding methods that balance efficiency and performance.

In this study, we propose MTDP, a **M**ulti-**T**eacher **D**istillation approach for **P**rotein embedding, which aims to enhance efficiency while preserving high-resolution representations. By leveraging the knowledge of multiple pre-trained protein embedding models, the student model in MTDP learns a compact and informative representation of proteins. It takes amino acid sequences as input and generates embeddings that encode biologically meaningful features of protein structure and function. We evaluated our model in various scenarios and benchmarked it against widely used large protein embedding models. Our results demonstrate that the student model in MTDP achieves state-of-the-art performance while significantly reducing computational costs and resource requirements.

## 2 Methods and materials

Knowledge distillation is a training method used to transfer knowledge from a large, pre-trained model (the teacher) to a small, efficient model (the student). This approach can significantly reduce computational costs and memory requirements, as many parameters in deep neural networks can be redundant or even ineffective (Gromov *et al.* 2024). Recently, researchers have applied knowledge distillation to large protein language models to improve efficiency in protein embedding. However, traditional knowledge distillation methods often face two significant challenges. First, the student model performance typically fails to match the teacher model’s performance. For example, DistilProtBert (Geffen *et al.* 2022), a distilled protein language model, has shown promise in protein embedding but still struggles to match the performance of its teacher models. Second, selecting a suitable teacher model can be a daunting task. As different teacher models may excel in different aspects of protein representation, the choice of teacher models may be a key factor that leads to inferior performance of the student model.

To address these challenges, we propose MTDP, a multi-teacher distillation approach that leverages the strengths of multiple pre-trained protein language models. During training, MTDP adaptively selects the most informative teachers for each training sample, allowing the student model to learn a more comprehensive representation of proteins. By aggregating and distilling the knowledge from multiple teachers, this approach yields a more accurate and efficient protein embedding method. In the following section, we provide a detailed description of MTDP and outline the datasets used for pre-training and fine-tuning our model.

### 2.1 Model structure

The MTDP leverages the Evolutionary Scale Modeling (ESM) (Rives *et al.* 2021) and ProtTrans-series (Elnaggar *et al.* 2021) Transformers for distillation, which have demonstrated state-of-the-art performance in various protein-related analysis tasks. The student model in MTDP is a T5-based transformer model, well suited to handle various embedding processing tasks within a unified framework. Compared to models like bidirectional encoder representations from transformers (BERT) and XLNet, T5 offers flexibility in training methodologies and masking strategies, enabling the reconstruction of spans of tokens rather than single tokens. This makes it particularly suitable for capturing dependencies between amino acids. In our experiments, we used grid search to tune the number of layers used in the student model. Based on our experiments, the six-layer T5 student model is the most cost-effective choice. Following the trend that larger models tend to perform better, we select ESM2-33 and ProtT5-XL-UniRef50 as our teacher models, which are the largest models that can be run on a 24 GB commercial GPU, commonly found in workstations and medium-level high-performance computing systems. In particular, the student model in MTDP has a significantly smaller parameter scale (∼20 million) compared to ESM2-33 (∼650 million) and ProtT5-XL-UniRef50 (∼120 million), approximately 3% and 1.6% of their sizes, respectively.

### 2.2 Training strategy and application usage

#### 2.2.1 Pre-training the student model

**Figure 1.**
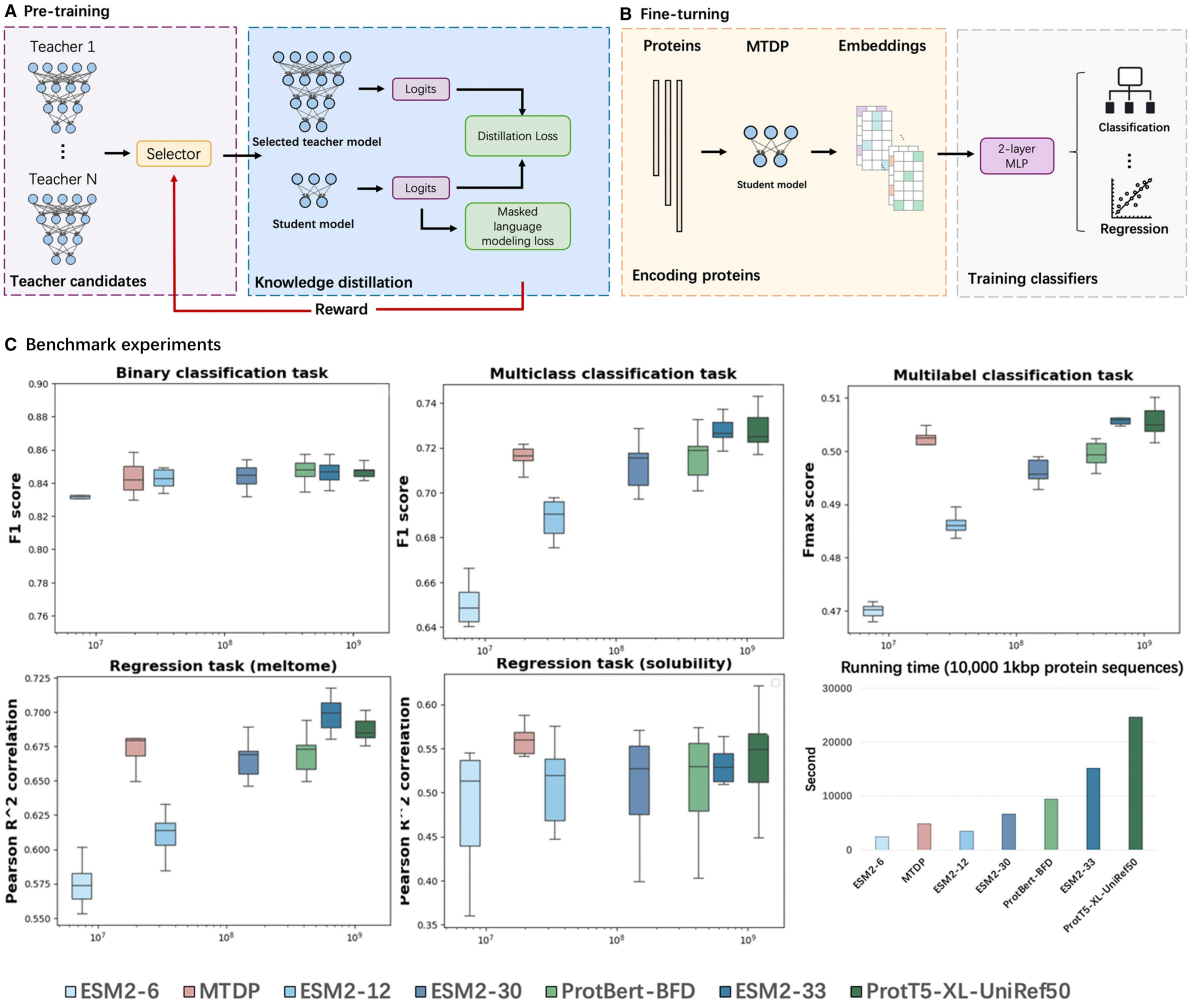
(**A**) The framework of multi-teacher distillation; (**B**) extracting protein embeddings using the student model in MTDP and use the embeddings to fine-tuning classifiers; and (**C**) the benchmark experiments on a wide range of protein-related tasks. *Y*-axis: the metric in each task. *X*-axis: the number of parameters in the model. We benchmarked the MTDP student model with the state-of-the-art tools, including ESM-series and ProtTrans-series large foundation model.

As illustrated in Fig. 1A, during pre-training, MTDP employs an adaptive teacher selection mechanism for knowledge distillation when pre-training. This is achieved through a reinforcement learning framework that learns a policy to select the optimal teacher model for each sample on an instance-by-instance basis during knowledge distillation. Instead of fixed weights, it considers features like input representation, teacher predictions, and losses for each instance to determine teacher selection. This process employs a logistic function to output a probability (weight) for each teacher, which is then sampled for the final selection. The goal is to optimize the performance of the student model through adaptive teacher selection. Because the scheduling policy used in our paper is a standard algorithm used in machine learning, we refer the reader to (Yuan *et al.* 2021) for a more detailed explanation of this reinforcement learning framework.

The MTDP student model is pre-trained using the masked language modeling task, where a random subset of amino acids in the protein sequence is masked, and the model predicts the original amino acids. The training objective consists of two components: the masked language modeling loss, which measures the difference between the predicted and original amino acids, and the distillation loss, which measures the difference between the student model’s embedded vectors and the teacher model’s embedded vectors. The distillation loss is calculated using the Kullback-Leibler (KL) divergence, which encourages the student model to mimic the behavior of the teacher models. The equations for calculating the losses are listed in Equation (1) (masked language modeling loss) and Equation (2) (distillation loss).

(1) $$L_{\mathrm{MLM}}=-\sum_{i=1}^{N}\sum_{j=1}^{V}I(y_{i}=j)\;\text{log}\;p(x_{i}=j)$$

where *N* is the sequence length; *V* is the vocabulary size; *y_i_* is the original amino acid at position *i*; *x_i_* is the predicted amino acid at position *i*; and I(·) is the indicator function.

(2) $$L_{\mathrm{Distill}}=\sum_{i=1}^{N}D_{KL}(p_{T}(x_{i})||p_{S}(x_{i}))$$

where $p_{T}(x_{i})$ is the output probability distribution of the teacher model at position *i*; $p_{S}(x_{i})$ is the output probability distribution of the student model at position *i*; and DKL(·|·) is the KL divergence.

We set a hyperparameter *α* to balance between these two losses, as shown in Equation (3). In our experiments, α=0.2 performs best. The reason of distillation loss having a higher weight maybe because the softened outputs of a larger, well-trained teacher model fits to train a smaller student model as demonstrated in (Hinton *et al.* 2015).

(3) $$L=\alpha L_{\mathrm{MLM}}+(1-\alpha )L_{\mathrm{Distill}}$$

By jointly optimizing these two losses, our MTDP approach enables the student model to learn a compact and informative representation of proteins, effectively leveraging the strengths of multiple teacher models.

##### 2.2.2 Fine-tuning classifier using the student model

After pre-training with the masked language task, the MTDP student model serves as a general protein language model similar to all the existing protein foundation model. Typically, query proteins are fed into the model to generate embeddings, which can then be used to fine-tune classifiers for downstream analysis tasks (Fig. 1B). As mentioned in Section 2.1, the student model uses significantly fewer parameters than the large foundation model, allowing for more efficient generation of these representations.

### 2.3 Data

#### 2.3.1 Pre-training data

MTDP is pre-trained on ∼500 000 proteins from UniProtKB (Swiss-Prot) provided by the teacher model (Elnaggar *et al.* 2021). To prevent excessive memory usage, we followed the design of other protein embedding models and set 1000 as the maximum length. Specifically, if the proteins exceed 1000 amino acids in length, we only consider the first 1000 amino acids. We employ an offline training strategy, utilizing ESM2-33 and ProtT5-XL-UniRef50 to generate protein embeddings on the same data with provided parameters. The advantage of offline training is that the teacher model is fixed and is only used to generate outputs/logits for the ∼500 000 proteins. Then, the pre-training process of the MTDP student model will be carried out with the guidance of these output embeddings. The MTDP is pre-trained on the masked language task with a masking probability of 15%. We conducted the pre-training process on four RTX 3080 24 GB Nvidia GPUs, with a batch size of 16. We use the AdamW optimizer with a learning rate of 3e-4. The model is trained for ten epochs using mixed precision with a warm-up ratio of 0.1. To demonstrate the advantage of multi-teacher distillation, we also train two distillation models, each using only ESM2-33 or ProtT5-XL-UniRef50 as the teacher, and compare their performance with our student model.

#### 2.3.2 Fine-tuning data

We evaluate the student model in MTDP on several benchmark tasks, with dataset information summarized as follows. All these datasets are widely used for the evaluation of the protein embedding model (Elnaggar *et al.* 2021, Rives *et al.* 2021, Outeiral and Deane 2024), and detailed information about the datasets can be found in the Supplementary File.

- Binary classification: classify proteins into membrane-bound or water-soluble.
- Multiclass classification: classify proteins into ten classes of subcellular localization.
- Multi-label classification: protein function prediction with gene ontology.
- Regression task: predict protein stability using (i) melting temperatures and (ii) solubility databases.

As shown in Fig. 1B, during fine-tuning, we follow the standard setting and apply global average pooling to the student model’s output to generate per-protein embeddings. Then, for each task, we feed these embeddings into a simple two-layer multilayer perceptron (MLP) and minimize the loss using the AdamW optimizer. We employ cross-entropy loss for classification tasks and mean squared error for regression tasks.

## 3 Result

We benchmarked the student model in MTDP against six commonly used protein language models from the ESM-series (ESM2-6, ESM2-12, ESM2-30, and ESM2-33) and ProtTrans-series (ProtBert-BFD and ProtT5-XL-UniRef50). We selected these models because they can at least encode a single protein at a time on a 24 GB GPU unit, making them suitable for comparison with our model. In the ProtTrans-series, there are also models with the same structure but trained on different datasets. In this case, we chose the best-performing model reported in their paper (Elnaggar *et al.* 2021) as the benchmark model.

In each task, we employed 10-fold cross-validation, recording performance metrics to generate box plots. Detailed descriptions of the metrics for each task are available in the Supplementary File. Additionally, we documented the model size and running time for each model to aid in comparative analysis.

### 3.1 Evaluation tasks

As shown in Fig. 1C, by distilling knowledge from the teacher models, our MTDP approach learns a compact and informative representation of proteins, striking a balance between performance and efficiency. The results reveal that the student model achieves nearly the same performance as its teachers, ESM2-33 and ProtT5-XL-UniRef50, across different tasks. Moreover, our model even outperforms its teachers in some tasks. To further measure the difference of the performance, we calculate the *P*-value between the student model and its teacher models. The results shown in Supplementary Table S1 reveal that MTDP framework successfully distill the knowledge from the teacher models.

Furthermore, we designed an ablation study to illustrate the effect of leveraging multiple teachers for knowledge distillation. The results shown in Supplementary Fig. S1 demonstrate that MTDP’s performance is significantly higher than that of a student model trained with a single teacher, highlighting the benefits of the multi-teacher distillation framework.

### 3.2 Efficiency of MTDP student model

Rapid analysis and interpretation of protein sequences are crucial, particularly when dealing with large-scale protein data. In our experiments, we ran all models on a single RTX 3080 24 GB Nvidia GPU and recorded the running time for processing 10 000 1 kb protein sequences. The results demonstrate that the student model in MTDP efficiently encodes protein sequences with minimal impact on performance. Additionally, it is noteworthy that MTDP, ESM2-6, and ESM2-12 can be deployed on smaller GPU memories (e.g. RTX 2080Ti 11 GB), thereby expanding their range of usage scenarios.

## 4 Discussion

In this paper, we present MTDP, an accurate and efficient protein embedding method. We demonstrate the reliability of MTDP in various protein-related analysis scenarios. Notably, the student model in MTDP achieves high-resolution protein embeddings comparable to those of large protein language models while significantly reducing computational costs and resource requirements, making it a promising approach for large-scale protein analysis.

Although MTDP has significantly improved protein embedding, there are still some limitations. In particular, the current version of MTDP is only trained on UniProtKB (Swiss-Prot), which is a small subset of the UniRef50 dataset widely used to train large models such as ESM2-33 and ProtT5-XL-UniRef50. To address this limitation, we plan to continuously update the UniRef50 and UniRef100 versions of MTDP in our GitHub repository. We will also provide MTDP framework as a developer mode to encourage users to choose their preferred teacher model for distillation.

## Supplementary Material

btae567_Supplementary_Data

## Data Availability

All data and codes used for this study are available online through: https://github.com/KennthShang/MTDP.
